# Some Points in Reparative Surgery of the Genital Tracts

**Published:** 1883-09

**Authors:** 


### SOME POINTS IN THE REPARATIVE SURGERY OF THE GENITAL TRACTS.

Dr. M. A. Pallen of New York, in a paper on this subject, writes: All fallings of the uterus, from the slightest prolapse to the completest procidentia, necessarily involve most or less folding of the vagina upon itself; and, should the substructure, the perineal conjunction, be absent, the process of vaginal folding ultimately becomes complete inversion. Without the necessary amount of time to properly discuss the relations of vaginal dislocations to the perfect integrity of the perinæum, I propose to formulate certain propositions.

1. Should there be perineal laceration, even if the uterine structure and circumuterine spaces be perfectly normal, the organ, sooner or later, necessarily sinks in the pelvis, most frequently in retroversion.

2. All perineal lacerations, from a simple submucous muscular sundering (of the *transversus perinæi*, *sphincter*, and *levatores ani* conjunctions), to a rent that extends into the bowel, necessarily beget vaginal dislocation, primarily as a slight, later as a complete rectocele, to be followed by a prolapse of the anterior wall, causing urethrocele and cystocele.

3. Urethocele and cystocele seldom occur spontaneously; they ensue from pressure above (very rarely), or they follow from perineal sundering or laceration. I have never seen a case of cystocele, or even much urethrocele, that was not associated with some prolapse of the posterior vaginal wall.

4. All operative procedures for the *suspension of a prolapsed uterus must be directed mainly to the posterior vaginal wall*, because it arches upon the perinæum below and the uterus above, serving chiefly as a column of support. The anterior vaginal wall, being straight and shorter, serves rather for the support of the urethra and bladder, and being adherent to the pubo-vesical spaces, it prevents the full bladder from rolling the uterus in retroversion.

5. Operations restoring the integrity of the perinæum and posterior vaginal wall, usually develop symmetrical correlations of the canal. In cases of complete procidentia, a perinæum restored by plastic procedures which strengthen the recto-vaginal septum will eventuate in a permanent cure, a condition I have never seen in making operations confined strictly to the anterior vaginal wall.

These propositions assumed, I feel satisfied that very many successful issues of *perineo-vagino-plasty* prove that the theory upon which the operation was based is correct, viz., that the conjunction of the transversus perinæi, sphincter ani, pubo ischio-coccygeus, and levatores ani muscles, (described, but never actually demonstrated as the perinæum) is the true and correct foundation upon which the posterior vaginal wall rests, and that the *support rendered by the connective tissue in front of the rectum is but secondary, in consequence of the variable calibre of the bowel.* The anterior column of the vagina is straighter and shorter, and, as before said, mainly supports the bladder and urethra; but the posterior vaginal column, added to the masses of blood-vessels furrowing the peri-vaginal connective tissue, tends to support the uterus; therefore, when the basement support of the vagina (perinæum) gives away, it folds down upon itself, and drags the uterus in retroversion. I would state *en passant*, that I exceedingly doubt the efficacy of the so-called ligamentous support of the uterus, farther than the misnamed structures (broad ligaments) serve as vehicles for carrying masses of erectile tissue and blood-vessels; and that in the healthy female the uterine body maintains its normal plane, or it is lifted, or it is depressed therefrom, in consequence of plentitude or emptiness of these same blood-vessels. Furthermore, I am disposed to think that all misplacements, except from direct or mechanical causes, depend upon fracture or destruction of the connective tissue in the circumuterine spaces, because of pathological changes in the blood-vessels.—*British Medical Journal.*

